# A predictive language model for SARS-CoV-2 evolution

**DOI:** 10.1038/s41392-024-02066-x

**Published:** 2024-12-23

**Authors:** Enhao Ma, Xuan Guo, Mingda Hu, Penghua Wang, Xin Wang, Congwen Wei, Gong Cheng

**Affiliations:** 1 School of Basic Medical Science, Tsinghua University, 30 Shuangqing Rd., Haidian District, Beijing, 100084 China; 2 Institute of Infectious Diseases, Shenzhen Bay Laboratory, Guangqiao Rd., Guangming District, Shenzhen, Guangdong 518000 China; 3 Beijing Institute of Biotechnology, 20 Dongdajie, Fengtai District, Beijing, 100071 China; 4 Department of Immunology, School of Medicine, University of Connecticut Health Center, Farmington, CT 06030 USA

**Keywords:** Predictive medicine, Microbiology, Infectious diseases

## Abstract

Modeling and predicting mutations are critical for COVID-19 and similar pandemic preparedness. However, existing predictive models have yet to integrate the regularity and randomness of viral mutations with minimal data requirements. Here, we develop a non-demanding language model utilizing both regularity and randomness to predict candidate SARS-CoV-2 variants and mutations that might prevail. We constructed the “grammatical frameworks” of the available S1 sequences for dimension reduction and semantic representation to grasp the model’s latent regularity. The mutational profile, defined as the frequency of mutations, was introduced into the model to incorporate randomness. With this model, we successfully identified and validated several variants with significantly enhanced viral infectivity and immune evasion by wet-lab experiments. By inputting the sequence data from three different time points, we detected circulating strains or vital mutations for XBB.1.16, EG.5, JN.1, and BA.2.86 strains before their emergence. In addition, our results also predicted the previously unknown variants that may cause future epidemics. With both the data validation and experiment evidence, our study represents a fast-responding, concise, and promising language model, potentially generalizable to other viral pathogens, to forecast viral evolution and detect crucial hot mutation spots, thus warning the emerging variants that might raise public health concern.

## Introduction

Severe acute respiratory syndrome coronavirus 2 (SARS-CoV-2) has sparked waves of coronavirus disease 2019 (COVID-19) pandemics, posing an ongoing threat to global health.^1,2^ Despite the persistent effort to develop vaccines, antiviral drugs, and antibody therapies, a significant obstacle for current COVID-19 pharmaceutical interventions arises from the rapid mutations in SARS-CoV-2 proteins, especially the spike (S) protein.^3–5^ Multiple mutations in SARS-CoV-2 proteins emerged as the pandemic progressed,^6^ some of which increased the virus’s binding affinity to ACE2 and evade immunity.^7–11^ The viral protein mutations result in the evolution of the virus, thereby partly driving the spread of successive pandemic waves.^12–15^ As a highly concerning lineage, Omicron emerged in November 2021, and its subvariant BA.1 rapidly substituted the prevailing Delta strains.^16^ Subsequently, multiple Omicron subvariants, including BA.2, BA.4, BA.5, BQ.1, XBB, CH1.1, EG.5, JN.1, KP.2, KP.3, XDV.1, and LN.1 promptly rise into the stage of the world, developing even stronger host immune evasion properties.^2,17,18^ Given the ongoing mutations in SARS-CoV-2 and the presence of several VOI (variants of interest), the ability to predict key mutations that contribute to immune escape and viral infectivity is of utmost importance for disease prevention.

Biomedicine is undergoing a revolutionary change, thanks to the technological advancements of artificial intelligence. Studies have represented the available viral sequences as letter strings^19^ and assessed the grammar and semantic fitness of the present sequences using natural language processing (NLP).^20–23^ For instance, Hie et al. have demonstrated that the same principles used to train a language model on a sequence of English words can also be applied to a sequence of amino acids. As a mutant virus must retain its infectivity and evolutionary fitness to escape, it must adhere to a “grammar” of biological rules.^21^ In a similar vein, we analogize the protein motifs and domains to human languages, such as words, phrases, and sentences, for modeling analysis and prediction. Like all other species, though mutation occurs inevitably, proteins in viruses like SARS-CoV-2 still have their more conservative sites and less conservative sites. By analyzing the amino acid features of mutation occurrence, some studies predicted the mutations that preserve infectivity and fitness, potentially revealing the mutations with more prevalence.^6,24^ For instance, all Omicron subvariants, including the most recent JN.1 and EG.5, possess the K417N substitution, which facilitates the virus to escape from humoral immunity,^9^ suggesting that this mutation may persist in future variants. Nevertheless, mutations also occur in a random pattern,^25^ resulting in specific mutations in a short period. The F486V, K444T, and F456L mutations, for example, were rarely found in BA.1, BA.2, BA.4, and BA.5 Omicron subvariants but emerged rapidly in the subsequent prevalent subvariants (e.g., BQ.1 and BQ.1.1).^26^

Given the limitations of current studies in predicting mutations based solely on present viral sequences,^6^ our research designs a delicate language model, named the semantic model for variants evolution prediction (SVEP), incorporating both the conservative regularity and unconservative randomness of combinatorial mutations to forecast the sequences of upcoming SARS-CoV-2 variants. It allows us to forecast the sequences of upcoming SARS-CoV-2 variants without the need for information on phylogenetic trees, deep mutational scanning (DMS), or 3D protein structure. We then validated our predictions using an HIV-1 pseudovirus assay incorporating SARS-CoV-2 S protein.

Two major barriers to eliminating or alleviating the continuous explosion of COVID-19 and other viral pandemics are pathogens’ nature of constant mutation and the prolonged time consumption of vaccine development. The latter causes the updated vaccine to be unable to catch up with the viral mutation rate. Therefore, predicting the potential variants that would prevail is crucial for the vaccines to keep pace with the viral mutation, similar to the “Red Queen Hypothesis.” The structure of our model significantly enhances data processing efficiency and reduces the consumption of computational resources, enabling the model to more effectively simulate combinatorial mutations. Therefore, our model has a unique advantage for predicting the emerging variants in a timely manner, aiding the fast response of vaccine development. Moreover, the results of this study provide potential insights into future SARS-CoV-2 variants, thereby significantly contributing to the development of COVID-19 interventions and potentially extending to other potential pandemics.

## Results

### Establishing a language model for the combinatorial mutations in SARS-CoV-2 Omicron sequences

**Fig. 1:**
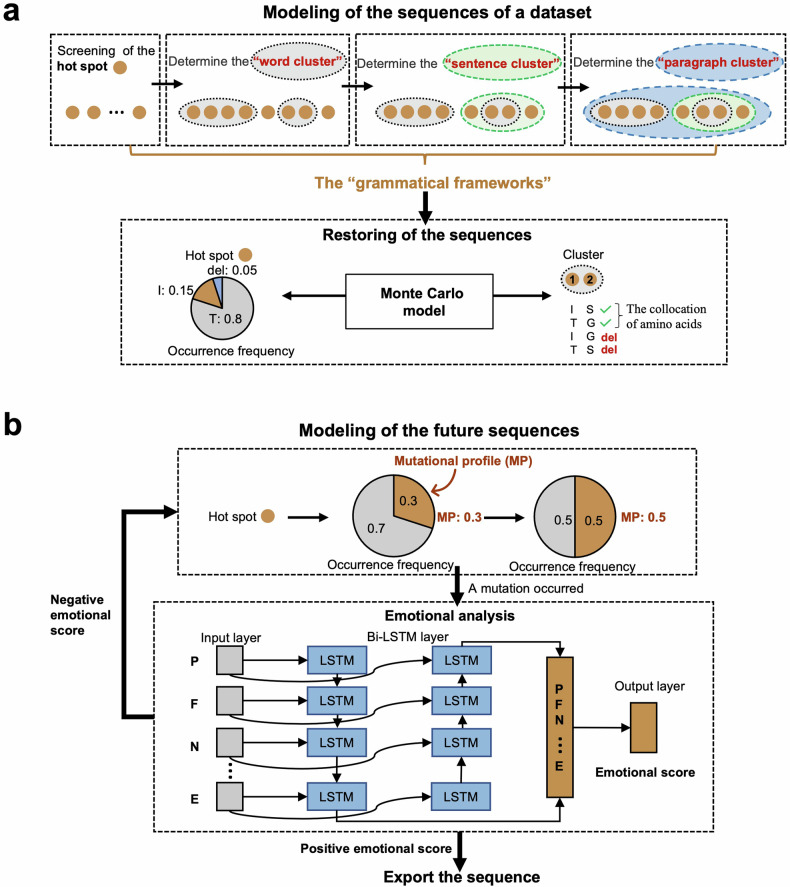
Modeling the combinatorial mutations in SARS-CoV-2 Omicron sequences. **a** The schematic diagram for determining the “grammatical frameworks” and modeling the sequences in dataset-1 based on the “grammatical frameworks”. The orange solid circles denote the hot spots. The black, green, and blue dashed circles denote the “word clusters,” “sentence clusters,” and “paragraph clusters,” respectively, and they formed the so-called “grammatical framework.” Monte Carlo simulation simulates the residues at each hot spot with the constraint of collocation of amino acids. For example, as the combination of amino acids I and G had never appeared together in one cluster, we would exclude the possibility of this collocation. **b** The schematic diagram for modeling the future sequences. Mutational profile is introduced to change the occurrence frequency of mutation at hot spots (for example, change the occurrence frequency of mutation from 0.3 to 0.5). Bi-LSTM language models are used for screening to exclude the generated sequences with low fitness based on the prior data. The input data were the amino acids within a “paragraph cluster”. The output layer exports a positive or negative score of the amino acid cluster, and only the sequences with positive scores are outputted

There are mainly three main steps for our model construction, which are the derivation of “grammatical framework” to construct data’s regularity, the introduction of the “mutational profile” to incorporate random mutations, and a screening model to exclude the sequences with minimal likelihood to emerge in the real-world. Initially, we collected the sequences of the S1 peptide in SARS-CoV-2 Omicron variants from April 15th, 2022, to September 15th, 2022, and then performed multiple sequence alignments. Interestingly, September 2022 is the time when the first omicron waves ended, and April 2022 is the time when the BA.2 wave is at its peak. The Omicron S1 sequence data from the 14th to the 685th residues^1^ for those five months were subsequently extracted as the dataset-1 (Supplementary Fig. 1a). Subsequently, to separate the conservative and unconservative part of the S protein and further reduce the data dimension space, we defined the stability of an amino acid site in S1 sequences by Three Days’ Frequency (TDF), which is the percentage of amino acid for a residue site within three successive days. The residue with the highest TDF was defined as the dominant residue of each residue site (Supplementary Fig. 2a). Compared to other definitions of conservation such as the phylogenic tree, the concept of TDF allows us to include the latent information into account just as other techniques, and it is much easier and faster than constructing the phylogenetic tree since we need a validated model for the tree construction which costs time. The sites exhibiting significant variation ( > 0.09) in the TDF of the dominant residue over time were identified as the “hot spots” (Fig. 1a and Supplementary Fig. 2b, c). As a result, we defined 84 hot spots and 588 non-hot spots in the S1 sequences of SARS-CoV-2 Omicron (Supplementary Fig. 2d). To reduce further the dimension of the hot spot data and mimic the natural human language, we decided to employ a clustering approach. We initially grouped the related hot spots, forming the “word clusters,” and subsequently grouped the “word clusters” into “sentence clusters” and “sentence clusters” into “paragraph clusters” (Fig. 1a and Supplementary Figs. 3–5). The “word clusters,” “sentence clusters,” and “paragraph clusters” comprised the so-called “grammatical frameworks” of dataset-1 (Fig. 1a and Supplementary Fig. 6). Noteworthy, “words,” “sequences,” and “paragraphs” structures could capture the long-range interaction between nucleic acids as they are defined and clustered by shared latent pattern but not the physical distance in the sequences. The frameworks served as a simplified and structured representation of the hot spot data, enabling us to analyze the regularity of the combinatorial mutations in the S1 sequences of the Omicron variant more effectively and intuitively. Thus, the model can grab the data’s latent pattern and the conservative information more efficiently from the variants’ evolution, easing the model’s processing time.

We construct the regularity based on the framework after deriving “grammatical frameworks” from the inputting dataset-1 (Fig. 1a and Supplementary Fig. 6). To simulate the amino acids at each hot spot, we employed the Monte Carlo (MC) simulation, known for generating random outcomes based on occurrence frequencies, to simulate the amino acids at each hot spot (Fig. 1a). In the dataset-1, we observed that each amino acid was frequently associated with a specific set of co-occurring amino acids within each cluster, forming the collocation of amino acids (Fig. 1a). This collocation is seen within each level of “word cluster,” “sentence cluster,” and “paragraph cluster”. The simulation is thus based on the constructed “framework” (See in Methods). To ensure that the generated amino acids align with the observed conditional frequency and adhere to the co-occurrence patterns in dataset-1, we constrained the collocations of amino acids within each cluster (Fig. 1a). For example, as the combination of amino acids I and G had never appeared together in one cluster, we would exclude the possibility of this collocation during the simulation. Considering co-occurrence patterns of mutations within the same cluster enhanced the accuracy of the generated amino acids within each cluster. By incorporating these constraints and utilizing the MC simulation technique, we grant our model with the regularity of combinatorial mutations (Fig. 1a).

Despite the regularity in viral evolution, combinatorial mutations also include random events, and without including the randomness, the model could only generate sequences that have already emerged. There needs to be more than just identifying the regularity pattern of viral evolution to build a prediction model. Thus, we aim to simulate the randomness of the mutations by introducing a new variable named mutational profile to generate combinatorial mutations (Fig. 1b). Mutational profile is defined as a variable that determines the frequency of mutations. It refers to the combined effect of all the mutational processes that resulted in the accumulation of the observed mutations, yet we might know what those latent effects are and how they interact to provide the final mutation frequency. The mutational profile drives the occurrence of random mutations and thus generates a wide variety of possible viral sequences that may or may not have appeared before. Subsequently, we assessed the evolutionary fitness of the combinatorial mutations to dataset-1 by a language model to identify and remove the sequences with lower fitness, that is, unlikely to be a natural sequence.^21^ For example, the sequence must obey the latent “grammar” of biological rules to achieve high fitness. The Bi-directional Long Short-Term Memory (Bi-LSTM) language model, capable of accessing the information forward and backward to capture more latent features compared to regular LSTM,^21^ was trained based on the inputting data, dataset-1 in that case (Fig. 1b). By inputting the amino acids, the Bi-LSTM model generated an emotional score representing how well the sequence aligns with the training data (Fig. 1b). The “paragraph clusters” with a negative emotional score, indicating lower fitness to the training data, were defined as sequences with “syntax errors” and removed as they were unlikely to represent the latent pattern of the viral evolution. This screening process allowed us to retain only the sequences with higher fitness, ensuring that the generated sequences adhered to the “grammatical frameworks” and accurately represented the regularity and randomness of combinatorial mutations. Thus, our evolutionary language model represents a potential tool for understanding the regularity and randomness of mutations.

### Validating the language model with existing SARS-CoV-2 variants and predicting potential future SARS-CoV-2 variants

**Fig. 2:**
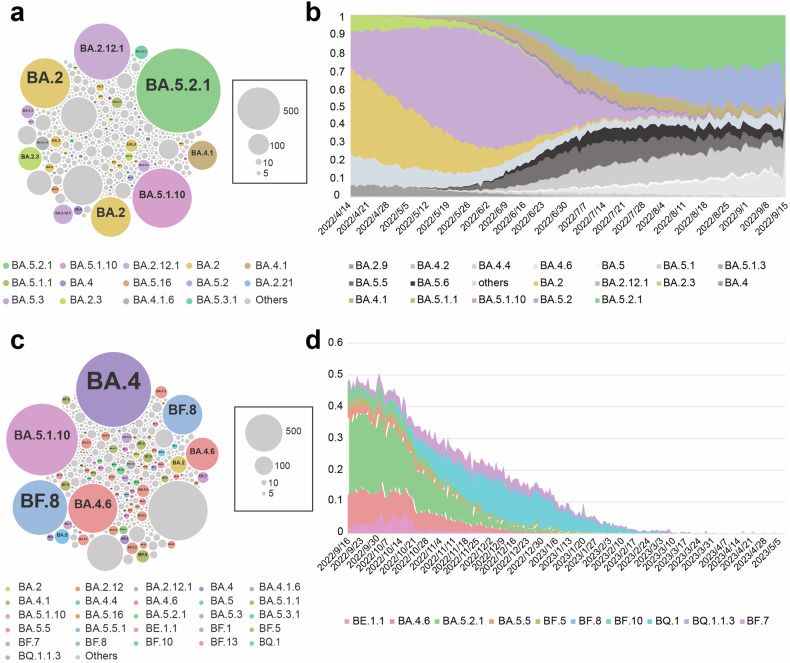
The validation of virus evolutionary language model and the prediction based on dataset-1. **a**, **b** The validation of the virus evolutionary language model without introducing a variable mutational profile. **a** 10,000 variants were generated by simulation based on the “grammatical framework,” and we checked whether the simulated variants could represent the variants of real data. The larger the circle, the more numbers of this variant are simulated, and each circle denotes a unique Omicron variant that might belong to the same lineage. **b** The schematic pattern of the prevailed variants during the timeframe of dataset-1 represents the real distribution pattern of the variants in that era. The variants belonging to the same lineage/sublineages are endowed with the same color in (**a**, **b**). **c**, **d** Using the evolutionary language model with a variable mutational profile to make predictions. **c** The variants were generated by 10,000 simulations using the model, but now mutational profile incurs random mutations. **d** The frequencies of the top 10 variants predicted by the model during the timeframe of dataset-2 (September 16th, 2022, to May 10th, 2023), right after the time period of input dataset-1. The models in (**a**–**d**) were developed based on dataset-1, and the prediction result is verified by the data in dataset-2

After establishing this evolutionary language model and before making the prediction, we need to conduct a comprehensive validation for our model using the available SARS-CoV-2 Omicron variants. The validation aims to determine whether our model could accurately represent the regularity of combinatorial mutations in sequences in dataset-1 after clustering into the “grammatical framework.” We wanted to check if we could simulate dataset-1 back using the “grammatical framework” after the dimension reduction to prove that our model retained most of the essential information of data. For this purpose, we performed 10,000 simulations at each hot spot using the model to generate sequences based on the framework built on dataset-1. During the validation process, the simulated sequences were compared to the actual variants observed in dataset-1 to evaluate how well the model-generated sequences aligned with the real-world viral sequences. The results showed that 67.7% of the simulated sequences matched the sequences in dataset-1, meaning only 32.3% of them did not match with any known lineages, indicating that the model successfully captured the underlying regularity of combinatorial mutations (Fig. 2a). For here, variants are not lineages as different variants might be included in the same lineage or sublineage. The identified variants, such as BA.5.2.1, BA.2.12.1, BA.2, BA.5.1.10, BA.4.1, and BA.2.3, were consistent with those observed in dataset-1 as they are also the dominant variants during the time-frame of dataset-1 (Fig. 2a, b and Supplementary Fig. 1a), further supporting the predicting capacity of this model. Moreover, the results showed that the top three simulated variants, BA.2.12.1, BA.5.2.1, and BA.2, were also the top three variants in the real dataset-1 (Fig. 2b and Supplementary Fig. 7a). This successful alignment between the simulated and observed variants in dataset-1 demonstrated that the virus evolutionary language model could accurately represent the regularity of combinatorial amino acid mutations present in the given dataset.

After validation, we conducted a retrospective study using only information available before the pandemic to make predictions. We thus evaluated whether our model could represent the randomness of combinatorial mutations by introducing and controlling the variable mutational profile, representing the occurrence frequency of mutation. That is, we want to examine the prediction capacity of our model. We still conducted 10,000 simulations at each hot spot, but now we changed their mutation frequency and used the Bi-LSTM language model for screening. This screening process is the best choice for our model after benchmarking (Supplementary Fig. 8). After we retrieved the simulated results using the model that only contained input data before September 16th, 2022 (dataset-1), we compared them with dataset-2, the actual S1 data of variants that emerged between September 16th, 2022, and May 10th, 2023 (Supplementary Fig. 1b). We collected the sequences of the S1 subunit in SARS-CoV-2 Omicron variants from that period and then performed multiple sequence alignments. After evaluating the precited sequences against the actual variants in dataset-2, we found that 70% of the simulated sequences could be assigned to specific Omicron subvariants, including BA.4, BA.5.1.10, BA.4.6, BA.5, BA.5.5, and BA.2 which emerge soon after the prediction (Fig. 2c). We can see, for the several variants we predicted most, which means they are the variants that are most likely to emerge according to our model, they take up about 50% proportion of all emerged variants during late 2022 (Fig. 2d). Additionally, we successfully predict the unseen BF, BE, and BQ subvariants, which were not present in dataset-1 but emerged after the timeframe of input data, is a significant validation of the predictive capabilities of our model (Fig. 2c and Supplementary Fig. 7b). The prediction of known and previously unknown subvariants indicated that our model could elucidate viral evolution and predict future SARS-CoV-2 variants. By introducing the mutational profile into the model, we significantly extended the ability to forecast the regularity and randomness in mutations, thereby indicating future viral variants.

### Updating the SARS-CoV-2 variant data for predicting potential SARS-CoV-2 variants by the language model

Predicting future SARS-CoV-2 variants or influential amino acids’ residue mutation is of significance as it allows more time to develop proactive responses at earlier stages of viral spread, potentially mitigating the impact of new variants on public health.^6^ Building upon the previous success of our language model of SARS-CoV-2 evolution in shedding light on viral evolution and accurately predicting future variants, we next aim to update our prediction for potential future variants based on the sequences that emerged between September 2022 and May 2023 (dataset-2) and between May 2023 and October 2023 (dataset-3). Previously, we first predicted the future variants based on dataset-1, yet it might be outdated for the present epidemic status. Repeating the prediction based on dataset-2 and dataset-3 would grant us insight into the near future’s viral evolution, providing a practical application for our model and also further validating the model’s integrity. We employed the exact modeling and validation approach used previously to accomplish the prediction.

**Fig. 3:**
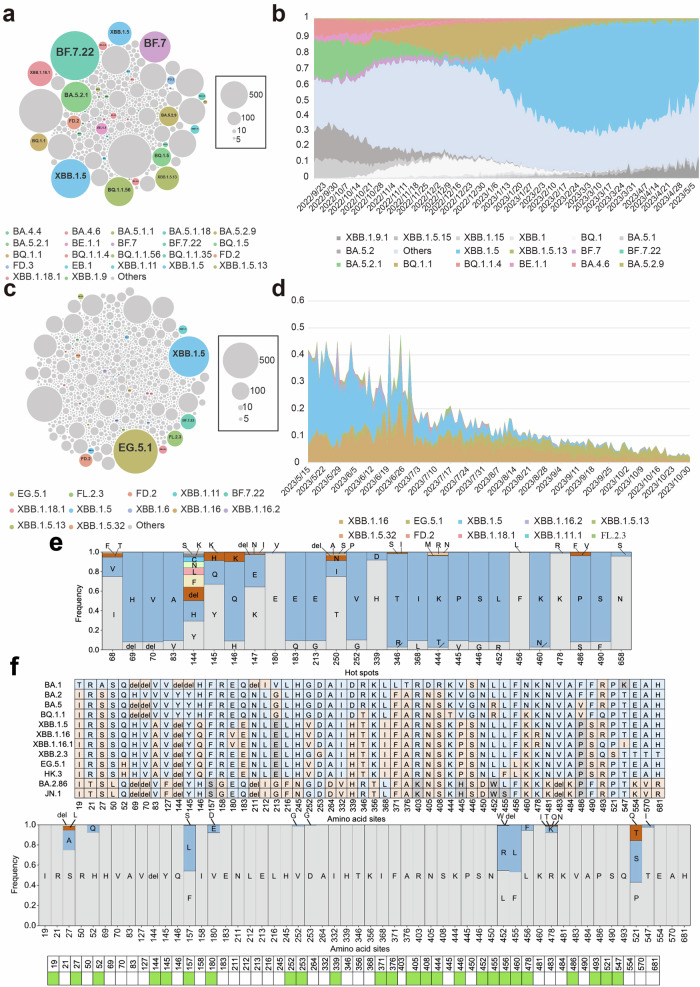
Updating the SARS-CoV-2 variant data for predicting potential future. **a**, **b** The validation of the virus evolutionary language model without introducing a variable mutational profile. **a** 10,000 variants were generated by simulation based on the “grammatical framework”. The larger the circle, the more numbers of this variant are simulated, and each circle denotes a unique Omicron variant. **b** The schematic pattern of the prevailed variants during the timeframe of dataset-2, representing the real distribution pattern of the variants in that era. The same variants are endowed with the same color in (**a**, **b**). **c**, **d** Using the evolutionary language model with a variable mutational profile to make the prediction. **c** The variants were generated by 10,000 simulations using the model but including mutational profile to incur random mutations. **d** The frequencies of the top 5 variants predicted by the model from May 11st, 2023, to July 1st, 2023, right after the time period of input dataset-2. **e** The frequencies of the amino acids at each hot spot generated by the model with a variable mutational profile. The models in (**a**–**d**) were developed based on dataset-2, and the prediction result is verified by the data after dataset-2. **f** The mutations in Omicron variants and the predicted results by the virus evolutionary language model based on dataset-3. **e**, **f** del denotes the amino acid deletion

To begin, we still conducted 10,000 simulations based on the constructed “grammatical framework” built by dataset-2 without introducing the mutational profile, thereby focusing on validating the regularity of the mutations. Similarly, we compared our simulated results against the variants present in the raw data (dataset-2) to assess how well the model-generated sequences aligned with the actual variants. Approximately 40% of the simulated sequences matched the variants observed in dataset-2. Notably, the majority of these matched sequences were XBB.1.5, BQ.1.1, BA.5.2.1, BA.4.6, and BF.7 subvariants (Fig. 3a). In both the simulated results and dataset-2, XBB.1.5 emerged as the most frequent Omicron lineage, followed by the BQ and BF subvariants (Fig. 3b and Supplementary Fig. 7c). We thus successfully simulate back the dataset-2 from the constructed “grammatical framework”. These results prove that our language model could effectively represent the regularity of the amino acid mutations present in the sequences in dataset-2, keeping the vital information after dimension reduction.

Likewise, after validation, we perform the prediction and test our predicted results against the variants that emerged after the timeframe of dataset-2. For this purpose, we collected the sequences of S1 peptide in SARS-CoV-2 Omicron variants from May 15th, 2023, to October 31st, 2023 (dataset-3), and then performed multiple sequence alignments (Supplementary Fig. 1c). We chose October 2023 as the end period of our dataset since it is time just before the emergence of current VOIs, JN.1 and BA.2,86. By comparing the predicted sequences based on dataset-2 with the actual sequence in dataset-3, the dataset including the sequence data after the period of dataset-2, we found that the XBB.1.5 Omicron subvariant was prevalent in both the simulated results and real data dataset-3 (Fig. 3c, d), and XBB.1.5 was known as the dominant strain for the summer of 2023. We found out that the top 5 variants predicted by our model based on dataset-2 take up about 40% proportion of all emerged variants from May 2023 to October 2023 (Fig. 3d). Furthermore, the model successfully simulated the emergence of previously unseen Omicron subvariants in dataset-3, including XBB.1.16, XBB.2.3, GB.2, FL.2.3, and EG.5 (Fig. 3c). XBB.1.16 and EG.5 later became the dominant strain and once VOC (now VOI).^2^ We successfully predict them before their emergence. The ability to predict Omicron subvariants not present in the training dataset (dataset-2) and emerged in the later real-world demonstrates the capacity of the model to forecast potential vital variants. To further investigate these predicted variants, we analyzed the amino acids at each hot spot for these unknown predicted variants (Fig. 3e). Notably, the frequency of some predicted mutations, such as E180V, V252G, and K478R, increased between May 16th, 2023, and July 1st, 2023 (Fig. 3d and Supplementary Fig. 9). These results validate the capacity of our predicted model for effectively predicting emerging mutations and variants.

As the COVID-19 pandemic turns into a new stage of co-existence, the evolution trend of the virus switches from disease severity into transmissibility. Several mutants with enhanced immune escape capacity become concerned, including BA.2.86 and JN.1. After examining our model from two different datasets extracted from two different time points, we have already proved and validated the efficacy and accuracy of our model. However, a most recent update is now required to provide a hint about the post-emergency stage. We thus ran our model for the third time but now on a more recent dataset-3, including the spike data before the emergence of either JN.1 or BA.2.86. As a result, we found that even though we did not predict the complete sequence of those two variants within the 10,000 output sequences, we did predict about half of all essential mutations for them, including D339H, G446S, L452W, and F486P (Fig. 3f). Consequently, before their emergence, we successfully predicted future concerned variants, including BF.7, BQ.1, XBB.1.16, and EG.5. For those variants that incur an extraordinary amount of mutation and unseen deletion or even insertion, which makes them hard to predict, our model still extracts most of their vital mutation. By introducing the mutational profile into the model, we simulate both the regularity and the randomness of the mutations.

### Predicted variants show significantly enhanced infectivity

**Fig. 4:**
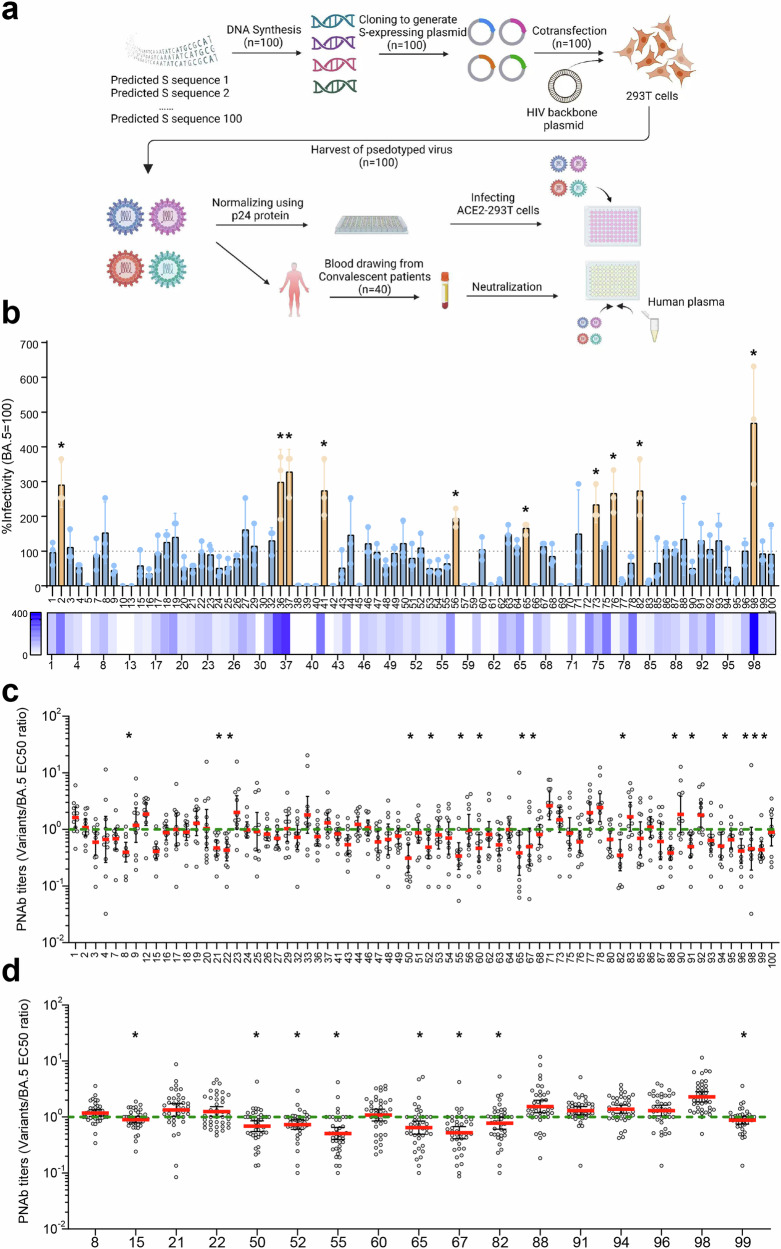
The experimental evaluation of the model-predicted SARS-CoV-2 spike variants. **a** The schematic diagram of the wet-lab experiments for plasmid synthesis, pseudoviruses construction, infectivity measurement, and neutralization assay. **b** The percent infectivity of predicted variants compared to that of BA.5 variant, which represented by TCID50 values. The TCID50 value of BA.5 is set as 100%. The PNAb titers measured by the relative ratio of EC50 values of predicted variants/BA.5 for 12 blood samples (**c**) and 40 blood samples (**d**). The EC50 value of BA.5 sequence is set as 1, and the measurement is geometric mean

To further validate the robustness of our model, we conducted a wet experiment, a crucial step to test its efficacy in real-world scenarios. We selected our first prediction from dataset-1 for our viral infectivity and immune evasion studies. Based on the prediction result of the first timeframe, we carefully handpicked the top 100 sequences that were most likely to emerge in the future for our further experimental evaluation and residue mutation analysis (Fig. 4a). We chose 100 sequences, each of which had been generated at least 5 times, a number we deemed sufficient for our model to identify them as potentially highly transmissible sequences. These 100 sequences, denoted with numbers from 1 to 100, were then synthesized and cloned accordingly to generate S-expressing plasmids. After the cloning and plasmid propagation in the *E. coli* system, 100 pseudoviruses for each 100 predicted S1 protein were constructed by plasmid transfection in HEK-293T cells (Fig. 4a). The liquid spectrometry measuring the binding affinity of ACE2 receptor protein revealed that most of the constructed pseudoviruses with low luciferase luminescence (RLU) value also corresponded with a low binding affinity (Supplementary Fig. 10). Excluding the pseudoviruses with extremely low RLU and binding affinity, 83 out of 100 pseudoviruses were deemed suitable for our infectivity study.

Before proceeding with the infectivity analysis, we implemented p24 quantification to precisely standardize the concentration of all pseudoviruses. This meticulous step was essential to ensure that the same amount of pseudoviruses were cocultured with ACE2 receptors for measurement. The 50% tissue culture infectious dose (TCID50) values for the infection assay were obtained and compared according to the Reed-Muench method for each pseudovirus. The results revealed 10 out of 83 pseudoviruses with sequence numbers #2, #36, #37, #41, #56, #65, #73, #76, #82, and #98 that exhibited significantly enhanced infectivity when compared with BA.5 sequences (Fig. 4b). Among them, variant #98 demonstrated the highest relative infectivity with an average level of 5-fold compared to that of BA.5. These precise results underscore the potential impact of our research in predicting variants with greater infectivity, the ones that are more likely to pose a threat in the future. The amino acid sequences of those 100 variants are included in the supplementary.

### Predicted variants exhibit increased immune evasion

Besides the viral infectivity of the predicted variants, another characteristic of concern that deserves further evaluation and relates to viral fitness is the neutralization escape of the variants. In our study, we recruited 48 volunteers for blood donation, all of whom shared a similar immune background of recovered patients vaccinated with a WHO-approved Ad5-vectored SARS-CoV-2 vaccine. We then performed a serum antibody neutralization assay to examine the immune evasion for each predicted sequence by coculturing the serum, pseudoviruses, and ACE2-expressing HEK-293T cells together (Fig. 4a). The pseudoviruses that failed to be neutralized by the anti-sera and infected more 293 T cells were the ones that desired more attention. The geometric mean of the ratios of each sequence to BA.5 for EC50 titer is thus set as a baseline control for comparison. In consequence, compared to that of the BA.5 variant, 15 potential variants were discovered with significantly lower half maximal effective concentration (EC50) value, including sequences #8, #21, #22, #50, #52, #55, #60, #65, #67, #82, #88, #91, #94, #96, and #98, yet those results were illustrated by preliminary studies adopting only 12 blood samples (Fig. 4c). When including all 40 eligible human blood samples, we discovered only 6 predicted variants #50, #52, #55, #65, #67, and #82 showed a significantly increased capacity of immune evasion (Fig. 4d). To be noticed, the difference in the immune evasion is below 2-fold, yet the difference is still significant. The immune escape capacities of variants #15 and #99 were also significantly higher for the latter case, yet their differences were below 1.3-fold.

Taking into account the sequence matching, we found that among those 6 sequences with enhanced immune evasion capacity, sequences #55 and #67 matched the lineages of BQ.1 and BQ.1.12, respectively. As BQ.1 and BQ.1.12 were dominant variants of late 2022, this finding further exemplified the accuracy and reliability of our prediction model. Interestingly, BQ.1 and BQ.1.12 variants were not included in our input dataset and only emerged after the construction of our model. These results verified that our model is able to predict not only enhanced infectivity but also immune evasion, providing a strong foundation for future research and understanding of SARS-CoV-2 variants.

### Predicted variants share vital mutations with circulating Omicron variants

**Fig. 5:**
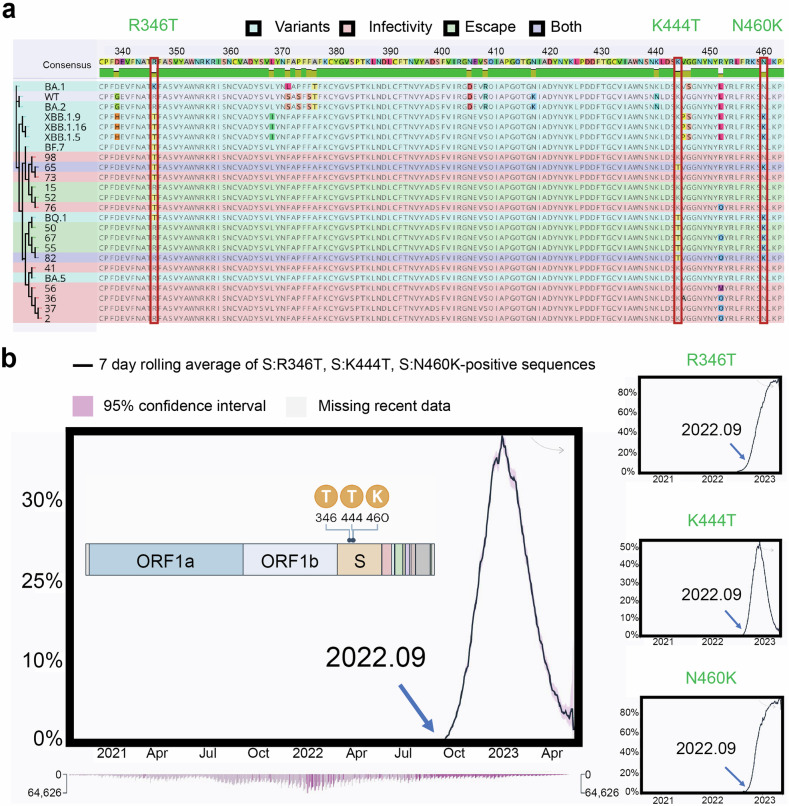
The residue mutation analysis of the concerned sequences outputted by the prediction model. **a** The multiple sequence alignment and phylogenetic tree of the 10 viral infectivity enhanced sequences and 6 immune evasion capacity increased sequences with R346T, K444T, and N460K residue mutations labelled. **b** Average daily prevalence of SARS-CoV-2 variants with labeling of residue mutation sites 346, 444, and 460

The previous evaluations on viral infectivity and immune evasion revealed several vital mutations in S1 sequence that might be related to viral fitness. As mentioned, we retrieved 10 predicted sequences of significantly enhanced infectivity and 6 sequences of increased immune evasion capacity. To further elucidate the evolutionary relationships and potential functional implications of SARS-CoV-2 variants, we constructed a comprehensive phylogenetic tree incorporating both prevalent and predicted variants (Fig. 5a). We found that sequences # 65 and #82 were included in both groups of immune escape and infectivity, illustrating their potential to emerge in the future. By analyzing the residue mutations that occurred in those variants, we discovered several common mutations, including R346T, K444T, N460K, R452Q, R685 deletion, and N658S. More precisely, we found that R346T, R685 deletion, L452Q, and N658S sites appeared more frequently in infectivity-enhancing sequences, while K444T and N460K were detected more often in antibody evasion-enhancing sequences. The structural analysis of the BA.5 S protein illustrated that most of them had only a minimum effect on the protein structure (Supplementary Fig. 11).

Among the residue mutations we detected above, some of them were consistent with previous findings, which exemplified their importance to viral evolution and thus might require greater attention. For instance, previous studies have reported R346T, K444T, and N460K as convergent mutations with enormous growth advantages and were mutated in at least five independent Omicron lineages, the major COVID-19 lineages since 2022.^27,28^ Besides residue spots 346, 444, and 460, residue spot 452 was also included in this list of important mutation sites,^28^ so the detection of R452Q also backed the reliability of our model. For our study, among 6 predicted variants with enhanced immune escape, 83.3% of them (5/6) presented K444T and N460K mutations. Similarly, for 10 predicted variants of significantly enhanced infectivity, 50% of them (5/10) adopted the R346T mutation. These findings illustrate that N460K and K444T mutations are highly correlated to the immune escape, and R346T mutation is correlated with viral infectivity. Importantly, our model, built before September 2022, was able to accurately predict future mutations of interest in the Omicron S protein (Fig. 5b), demonstrating its potential for future surveillance efforts. Interestingly, sequence #65 contained all 3 mutations of greatest interest, which deserve more attention in future surveillance of Omicron variants.

K444T is one of the major Spike mutations of interest for SARS-CoV-2 Omicron variants BQ.1 and CH1.1. Due to their tremendous impact on global health, WHO once escalated BQ.1 as one of the variants of interest (VOI) and CH1.1 as one of the variants under monitoring.^2^ As there are currently no SARS-CoV-2 variants meeting the VOC criteria, VOI and variants under monitoring (VUM) at their time are the ones that raise the most attention, underscoring the importance of detecting this mutation by our model. Moreover, residue mutations R346T and N460K are more important as they are not only major Spike mutations of interest for BQ.1 lineage but also for the XBB lineage, including variants XBB.1.9.1, XBB.1.15, and XBB.1.16 (Fig. 5a).

### Benchmarking shows the model’s unique advantages and comparable accuracy

The wet-lab verification might not be sufficient for validating the efficacy of our work. Without elaborate benchmarks and comparisons with other models, it is hard to exemplify the advantages and drawbacks of our model. In this case, we select three decent and respective works also focusing on SARS-CoV-2 evolution and a random generator for our comparison.

#### TEMPO

TEMPO, a transformer-based mutation prediction framework, is a fantastic model that utilizes phylogenetic tree-based sampling to capture the temporal information and transformer to predict the mutation probability of sites.^29^ There are similarities and differences in features between our model and TEMPO. For a common advantage, we could both predict mutation sites, and both models take the temporal information into account to increase the model’s accuracy. TEMPO has the unique advantage of including phylogenetic information in the model, which helps it understand what occurred throughout evolution,^29^ while our model defines “TDF” to incorporate the temporal variable. Our model, in such cases, provides an alternative choice for TEMPO and could be used in the initial stage of a pandemic when no phylogenetic information is available.

The most essential benchmark comparison is the comparison of performance. As the available dataset for TEMPO and our training data for our first prediction is about to be in a similar timeframe, we thus decided to compare the prediction sites generated by our first prediction and TEMPO. We successfully included 10 out of 16 high-probability (*p* ≥ 0.5) predicted mutation sites mentioned by TEMPO. This result is comparable to TEMPO’s own prediction of 12 out of 16.^29^ Furthermore, our model also identified several mutation sites not included by TEMPO, demonstrating its unique capabilities.

#### Brian’s LSTM

Based on two major components, grammar (or syntax) and meaning (or semantics), Hie et al. constructed an unsupervised NLP model to predict the viral escape capacity for different mutations and used three different viruses to examine the model’s validity.^21^ As outputs, this model could give out two scores for each mutation combination (semantic and grammar). The “semantic” component refers to the amplitude (or extent) of the change of the viral sequence, while the “grammar” component refers to the viral fitness. The mutations and the combinatorial mutations with high semantic scores represent a large shift from the original viral sequence, and the high grammar score represents high fitness resulting from the mutations. Hie believes that the mutations that score high for both components are likely to be the mutations that lead to immune escape.

To compare our model with Brian’s model, we used Brian’s model to test the mutation combinations of the top 10,000 sequences predicted from dataset-1 (which we believe to be the top 10,000 variants that are likely to emerge after the time dataset-1 is collected). As a result, we found 9566/10,000 (95.66%) of the sequences have higher semantic scores, 6388 (63.88%) of the sequences have higher grammar scores, and 6,034 (60.34%) of them have both higher semantic and grammar scores when compared to the dominant variants in the timeframe of the input data (dataset-1). This result helps to validate that our model is consistent with Brian’s LSTM model, and the essential variants predicted by our model are also the concerned variants for Brian’s model.

#### EVEscape

EVEscape, with its unique combination of deep learning and fitness predictions, quantifies the viral escape potential of given mutations at scale.^30^ Its distinct advantage over other models is its independence from information from surveillance sequencing, experimental scans, or three-dimensional structures of antibodies to make predictions.^30^ This advantage is particularly crucial in providing an early warning time critical for vaccine development. Likewise, our model, operating as an early alarm for potentially prevailing variants, could significantly impact vaccine development by making predictions solely based on sequence data.

**Fig. 6:**
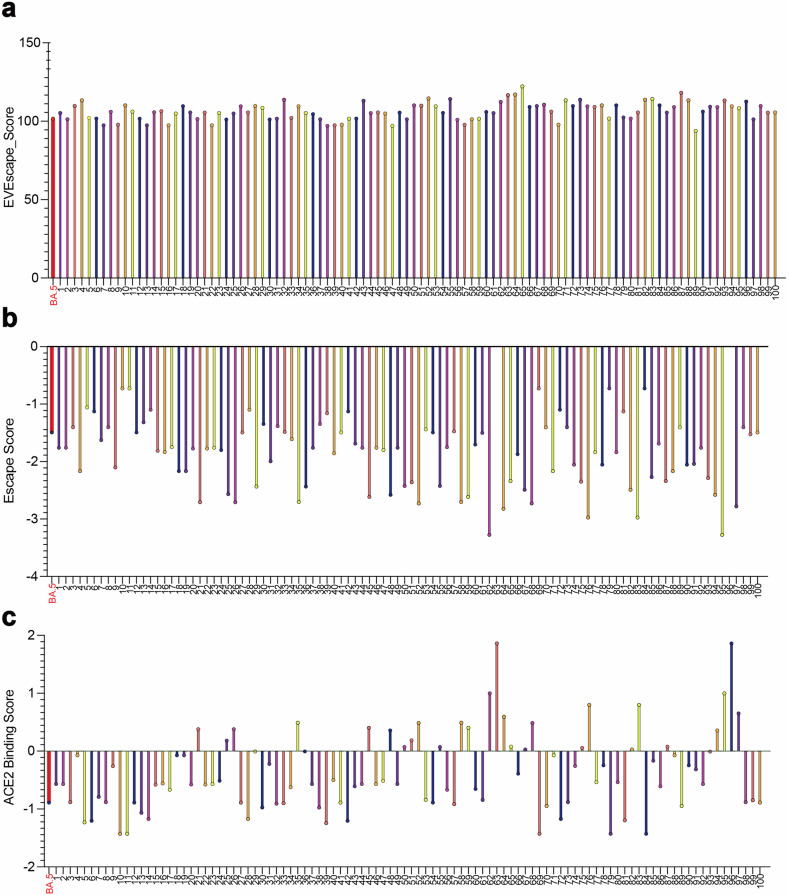
The comparison of the predicted results with other models. **a** EVEscape’s escape score of the 100 predicted sequences from our model. The higher value represents the higher immune evasion capacity. **b** MLAEP’s escape score of the 100 predicted sequences from our model. The lower value represents the higher immune evasion capacity. **c** MLAEP’s ACE2 binding score of the 100 predicted sequences from our model. The higher value represents the higher ACE2 affinity. The 100 sequences predicted by our model in (**a**–**c**) are based on dataset-1, and the dominant variant BA.5 (marked red) during the era of dataset-1 is used for comparison

As EVEscape does not generate new sequences or mutation sites but predicts the immune escape potential and antibody affinity for a given mutation, we could not compare our model with EVEscape directly. Instead, we tested the top 100 sequences predicted from dataset-1 and dataset-2 using EVEscape. Our model believed those sequences to have high immune evasion and low antibody affinity. As a result, 79% of sequences predicted from dataset-1 and 81% of sequences predicted from dataset-2 surpass the dominant variants in the timeframe of that dataset in immune escape capacity (Fig. 6a and Supplementary Fig. 12). For further verification, we increase the samples to the top 10,000 sequences and the consistent result is shown. About 65% of the sequences score higher than the prevailing strain at that period. This alignment with EVEscape further validates the accuracy of our model, instilling confidence in its predictive capabilities.

#### MLAEP

The Machine Learning-guided Antigenic Evolution Prediction (MLAEP) is a novel approach that combines “structure modeling, multi-task learning, and genetic algorithms” to predict viral immune escape and antibody affinity.^31^ This unique model, similar in function to EVEscape, provides a score of immune evasion and, uniquely, a score of antibody affinity. By leveraging the existing RBD region sequence information and Deep Mutation Scanning (DMS) dataset, MLAEP enhances its accuracy, albeit with the need for additional information.

Similarly, we input the top 100 predicted sequences into MLAEP to examine our model’s performance. These sequences represent the most likely variants that could emerge in the future. For the sequences predicted by dataset-1, 74% of our predicted sequence achieve a higher immune evasion score, and 71% of them achieve a higher ACE2 binding score than that of BA.2, BA.4, and BA.5, the dominant strain in the timeframe of our input dataset (Fig. 6b, c). Moreover, for the sequences predicted by dataset-2, 82% of them achieve a higher ACE2 affinity and immune escape capacity with the dominant strain XBB.1.5 of the timeframe for dataset-2. That is to say, our prediction is also consistent with MLAEP, so the sequences with warning potential predicted by us are also acknowledged by other models including Brian’s LSTM, MLAEP, and EVEscape.

Our model stands out with its unique advantage of extensive wet-lab validation, a testament to its reliability. Though both MLAEP and EVEscape included pseudovirus neutralization assays, the assays were not performed by the researchers themselves. Unlike the three mentioned models, which utilized only a few wet-lab data from other studies, we took the initiative to synthesize 100 generated sequences and construct pseudovirus assays (Supplementary Table 1). Each sequence was rigorously examined with 50 serum samples from recovered patients, ensuring the highest level of reliability in our results and conclusions.

Our model also circumvents the requirement of sophisticated, hard-to-obtain data such as phylogenetic trees, deep mutational scanning data, and protein 3D structure (Supplementary Table 2). By leveraging a “grammatical framework” for dimension reduction, our model operates efficiently with a relatively small amount of sequence data ( ~ 3 months’ data) and minimal computational power (personal desktop). A more intuitional description of the difference in computational burden between our models and the above models is that our model only needs CPU for prediction, yet all other models likely utilized GPU for prediction. This user-friendly approach empowers researchers with a fast-responding, pioneer method for early-stage pandemic warning.

#### Random generator

One last interesting comparison would be that against a random generator. We generated 100 sequences using the random generator and compared them with the top 100 predicted sequences from dataset-1. As we’ve seen, our 100 sequences contained several convergent mutations (R346T, K444T, and N460K) with significant growth advantages, observed in later emerging variants like BQ.1, CH1.1, XBB1.15, and XBB1.16. For our 100 predicted sequences, we found that 40 of them contained R346T, 8 contained K444T, and 6 contained N460K. In contrast, none of the 100 sequences generated by the random generator contained R346T or N460K, and only one contained K444T mutation, likely by chance (Supplementary Fig. 13). Importantly, our generated sequences included 10 out of 16 high-probability (*p* ≥ 0.5) predicted mutation sites that appeared in mid-2022, while the random generator’s predictions included none of them. These comparisons underscore the accuracy of our model and validate that our prediction results are not coincidental.

In conclusion, our model stands out when compared to TEMPO, EVEscape, Brian’s LSTM, and the random generator. It demonstrates several unique advantages and consistent accuracy, exemplifying the efficacy of the model.

## Discussion

The COVID-19 pandemic caused by SARS-CoV-2 continues to spread globally.^1,2^ Mutations in viral proteins occurred in a random pattern,^23^ yet studies have summarized the regularity of mutations using the available viral sequences. Recent studies employed natural language processing to model the viral escape^20,32^ and revealed the escape mutations as those that maintain viral infectivity but cause a virus to appear different to the immune system.^21^ A study defined and evaluated the scores for existing amino acid mutations in available viral sequences, revealing mutations that could contribute to future prevalent variants.^6^ In this study, we collected and determined the regularity of combinatorial mutations based on the SARS-CoV-2 S1 protein sequences in a given dataset. Nevertheless, some random mutations were rarely detected in the available viral sequences and arose rapidly over a short period. The low frequency of these mutations hindered the ability of modeling to predict these mutations.^6,33^ To overcome this problem, we introduced a mutational profile to the model to simulate the randomness of the mutations, thus allowing the model to simulate future variants.

Since the SARS-CoV-2 viral protein continues to undergo mutations, predicting future variants is crucial for developing treatments before the impending pandemic.^6,21^ We predicted the variants using our model based on different datasets collected from different time points and tested the results against the variants in datasets, including those that appeared after those time points. The results indicated that we successfully predicted variants including BQ.1, BF.7, BE.1.1, XBB.1.16, XBB.1.16.2, GB.2, FL.2.3, and EG.5 before their emergence. Natural recombination endows this variant with exclusive survival advantages, including enhanced transmissibility, additional Spike mutation and viral signature, and intensified antibody and immune evasion.^34,35^ During the process of this work, some of the predicted variants have caused epidemics and pandemics. The XBB.1.16 subvariant, for example, was prevalent in India by the end of March 2023 and showed enhanced immune evasion.^36–38^ In addition to these Omicron variants, our results predicted the previously unknown viral variants that may cause future COVID-19 pandemics. For example, EG.5 only appeared about 6 months after we made the prediction. For the variants that include too many mutations, deletions, and even insertions, such as BA.2.86 and JN.1, though we did not attain the variants themselves, we could still successfully predict the variants with D339H, G446S, L452W, and F486P mutations, the essential residue mutation for them. The model could be improved to include unseen deletion and insertion into consideration, which might allow us to predict variants such as JN.1 and BA.2.86 successfully. Our findings still verify our model’s ability to predict essential and somewhat conservative mutations for a highly unpredictable variant.

From our 100 predicted and synthesized Spike sequences, we retrieved 10 variants with significantly enhanced viral infectivity and 6 variants with significantly increased immune escape capacity. Moreover, we also detected that most of our 100 sequences had high binding affinity for hACE2 and infectivity. These results exemplify our model’s capacity to predict variants with relatively high vital fitness that may potentially emerge and become a concern in the near future. Overall, our study offers a virus evolution model to generate predictions for future SARS-CoV-2 variants and essential amino acid substitutions with only sequence information. Those features are rarely shown in other models, including TEMPO, MLAEP, and EVEscape. For TEMPO, phylogenetic information can be challenging to obtain as it requires vast amounts of sequence data and an accurate tree model trained from the data. By including “Three Days’ Frequency” (TDF) in model construction, our model also grabs the temporal information without needing phylogenetic trees. Moreover, our model can give out the specific type of mutation and hence could output the whole sequence instead of sites. For EVEscape, information is required in addition to mere sequences, such as 3D conformations (without antibodies). This conformation might not be easy to obtain by cryo-TEM or accurately predicted by a well-trained model with sufficient protein information. MLAEP utilizes the existing RBD region sequence information and Deep Mutation Scanning (DMS) dataset for the prediction, yet the experimental scan data such as DMS is also hard to attain, especially in the early stage of the pandemic.

As mentioned, our model is based merely on the sequence information and appeared mutation. Focusing on only the RBD region instead of the whole S1 subunit further increases the computational power and speed. In such cases, we sacrifice some of the prediction accuracy for the model’s simplicity. The model’s simplicity allows us to respond to viral outbreaks more swiftly and requires far less computational power and pioneer wet-lab experiments. As the significant mutation emerges much earlier before its prevalence, the increased computational speed allows us to update our model more timely and might identify the crucial mutation before its prevalence. The early warning of the potentially contagious variants provides insight for developing vaccines and answers the question of which sorts of antigen peptides the vaccines should target.

In conclusion, the low requirement of computational power and data types endows our model with the capacity to provide warnings in the early stage of a pandemic. Our model is thus timely and highly upgradeable, and the modeling results could offer insights into persistent COVID-19 pandemics and vaccine development. Hence, if sufficient sequence data is available, this model might also be generalized to predict mutations in other virus sequences. The focus on only hot spots undermines the predictive capability of our model, so in future studies, we will consider integrating a more dynamic approach to account for possible mutations in non-hot spot regions.

## Materials and Methods

### SARS-CoV-2 Omicron viral protein sequence dataset

The SARS-CoV-2 Omicron variant spike sequences collected from April 15th, 2022, to October 31st, 2023, were downloaded from the NCBI Virus Database (https://www.ncbi.nlm.nih.gov/labs/virus/vssi/). We only considered Spike sequences between 1173 and 1273 lengths. Multiple sequence alignment was subsequently performed by Muscle 5 (http://www.drive5.com/muscle/). S1 protein sequences from the 14th to the 685th residues^1^ were extracted as a dataset:

dataset-1: sequences collected from 2022.4.15 to 2022.9.15

dataset-2: sequences collected from 2022.9.16 to 2023.5.10

dataset-3: sequences collected from 2023.5.15 to 2023.10.31.

Datasets-1, dataset-2, and dataset-3 each have 441,083, 275,509, and 67,946 viral sequences. The sequences in dataset-1 belonged to BA.2, BA.4, BA.5, and their subvariants (Supplementary Fig. 1a). Most of the sequences in dataset-2 belonged to the BA.5, BQ, XBB, and their subvariants (Supplementary Fig. 1b). Most sequences in dataset-3 belonged to the FL, HV.1, XBB., EG. and their subvariants (Supplementary Fig. 1c).

### Modeling procedure

#### Determination of “grammatical frameworks” of a given dataset

Prior to commencing the modeling process, a crucial step involves establishing the “grammatical frameworks” inherent within the provided dataset. We defined the Three Days’ Frequency (TDF) of a specific as the percentage of this amino acid for a residue site within three successive days. Here the deletion was treated as an amino acid. The insertion of amino acids was not taken into account during the overall modeling process. The dominant residue of each site was defined as the amino acid with the highest TDF (Supplementary Figs. 2a, 14a, and 15a). Using the Pandas Python package,^39^ the TDF time series data of the dominant amino acid for each site of the sequences in dataset-1, dataset-2, and dataset-3 were calculated (Supplementary Figs. 2b, 14b, and 15b). Then we determined the “grammatical frameworks” of the hot spots in each dataset, as explained below.

1. **Screening for the hot spots**. The hot spots were defined as sites where the difference between the maximum TDF (*y*_max_) and the minimum TDF (*y*_min_) of the dominant residue was greater than 0.09 (*y*_max_ - *y*_min_ > 0.09) and where more than one amino acid appeared (Supplementary Figs. 2c, 14c, and 15c). Based on dataset-1, dataset-2, and dataset-3, 84, 26, and 30 hot spots were identified, respectively (Supplementary Figs. 2d, 14d, and 15d). At each hot spot, the maximum value of each TDF time series data was applied to standardize the time series data. Sites within the S1 protein that did not meet the criteria for designation as ‘hot spots’ were categorized as “non-hot spots” (Supplementary Figs. 2d, 14d, and 15d).
2. **Determination of the “word clusters.”** The “word clusters” were defined as the clusters of the TDF time series data with a similar trend. The TDF time series data of the dominant amino acid at different hot spots were clustered using the protocol reported in prior study.^40^ The hierarchical clustering built a hierarchy of clusters and yielded the number of clusters (*N*_c_) (Supplementary Figs. 3a, 16a, and 17a). The K-means cluster analysis divided the TDF data into *N*_c_ clusters, assigning each sample to the cluster with the closest mean TDF values. The K-means cluster results are shown in Supplementary Figs. 3b, 16b, and 17b. IBM SPSS Statistics 26 software was utilized to complete the above cluster analysis.
3. **Dimension reduction of the “word clusters” time series data**. The number of hot spots within the “word clusters” ranged from 1 to 39 (dataset-1), from 1 to 7 (dataset-2), and from 1 to 15 (dataset-3), respectively. The cluster analysis input data should be column vectors. In order to further cluster the “word clusters” into “sentence clusters”, the “word clusters” with multiple hot spots must be converted to column vectors. The principal component analysis (PCA)^41^ was employed to reduce the dimension of the TDF time series data within each “word cluster”. MATLAB R2020b (MathWorks, US) was used to perform the PCA. After PCA, the TDF time series data within each “word cluster” was reduced to a column vector. The maximum value in each column vector was then applied to standardize the “word clusters” time series data.
4. **Determination of the “sentence clusters.”** The “sentence clusters” were defined as the clusters of the “word clusters” time series data with a similar trend. The “word clusters” time series data were clustered by the hierarchical clustering and K-means approaches using IBM SPSS Statistics 26 software. The values of *N*_c_ detected by the hierarchical cluster approach for the three datasets were shown in Supplementary Figs. 4a, 18a, and 19a. Using the “word clusters” time series data and *N*_c_ as input data, the K-means cluster analysis was able to determine the “sentence clusters”.
5. **Dimension reduction of the “sentence clusters” time series data**. To further cluster the “sentence clusters” into “paragraph clusters”, the “sentence clusters” with multiple hot spots must be reduced to column vectors using PCA.^41^ MATLAB R2020b was applied to conduct the PCA. The maximum value of each column vector was then applied to the “sentence clusters” time series data in order to standardize it.
6. **Determination of the “paragraph clusters”**. The “paragraph clusters” were defined as the cluster of the “sentence clusters” time series data with a similar trend. The time series data of “sentence clusters” were clustered by the hierarchical and K-means approaches using IBM SPSS Statistics 26 software (Supplementary Figs. 5a, 20a, and 21a). The K-means cluster analysis was used to determine the “paragraph clusters”.

After the above cluster analysis, the dimensions of the TDF time series data of the hot spots in dataset-1, dataset-2, and dataset-3 were reduced. The “grammatical frameworks” of dataset-1 (Supplementary Fig. 6), dataset-2 (Supplementary Fig. 22) and dataset-3 (Supplementary Fig. 23) were compromised by the “word clusters,” “sentence clusters,” and “paragraph clusters”.

### Amino acid simulation based on the “grammatical frameworks”

The “grammatical frameworks” of dataset-1, dataset-2, and dataset-3 have divided the S1 protein sites into hot spots and non-hot spots and further reduced the dimension of hot spots into “word clusters,” “sentence clusters,” and “paragraph clusters.” We assumed that each non-hot spot conserved the dominant amino acid and that it could not change. Then we simulated the amino acids at the hot spots of the S1 protein based on the “grammatical frameworks.” The occurrence frequency of an event was defined by dividing the number of trials in which it occurred by the total number of trials. The Monte Carlo (MC) simulation approach can generate a variety of potential outcomes based on the occurrence frequency of an event. It is widely used in modeling the processes such as CO_2_ emission,^42^ the structure of proteins,^43^ and the structure of Zeolite.^44^ This paper employed the MC approach to simulate the amino acids at the hot spots. MATLAB R2020b was used to conduct the MC simulation (Supplementary Fig. 24), and its procedure is described in detail below.

1. **Simulation of the amino acids at each hot spot**. The mean TDF of an amino acid was determined by dividing the total TDF of an amino acid by the total number of TDF. Only amino acids with a mean TDF greater than 0.02 were considered in the simulation of amino acids at hot spots. The mean TDF was applied as the occurrence frequency to generate the amino acids at each hot spot (Supplementary Fig. 25). Here the deletion was treated as an amino acid. We used the randsrc function in MATLAB R2020b to generate the amino acids at each hot spot. The input data of the randsrc function were the dimension of the output matrix (1, 1), the amino acids, and the occurrence frequency of the amino acids: randsrc(1, 1, [double(‘amino acid 1’), double(‘amino acid 2’); the occurrence frequency of amino acid 1, the occurrence frequency of amino acid 2]). We defined the number of simulations num = 10,000. In each simulation, an amino acid was generated by the randsrc function at each hot spot.
2. **Constraint of the collocation of amino acids within each “word cluster”**. The prevalent residue at a hot spot was defined as the amino acid with a mean TDF over 0.2 (Supplementary Fig. 26). We observed that in each cluster, a prevalent residue was associated with a specific set of amino acids, which was defined as the collocation of amino acids (Supplementary Fig. 27). For instance, site 1 and site 2 constituted a “paragraph cluster”. For site 1, the prevalent residues were assumed to be I and T. Conditional frequency P(A | B) refers to the probability that event A will occur under the condition that event B has already occurred. P(site 2 = S|site 1 = I) and P(site 2 = G|site 1 = T) were greater than 0.9. Consequently, “IS” and “TG” were the collocations of amino acids for site 1 and site 2. When a prevalent residue was generated at a hot spot within a “word cluster”, the conditional frequency (CF) of the amino acids at other hot spots was calculated based on all datasets. The Pandas Python package^40^ was used to calculate the CF. The amino acids at other hot spots were subsequently constrained based on the CF by the randsrc function in MATLAB R2020b:
  If the amino acid at the hot spot 1 = I
  The amino acid at the hot spot 2 = randsrc(1, 1,[double(‘S’),double(‘G’); 0.9683, 0.0317])
  If the amino acid at the hot spot 1 = T
  The amino acid at the hot spot 2 = randsrc(1, 1,[double(‘S’),double(‘G’); 0.9097, 0.0903])
3. **Constraint of the collocation of “word clusters” within each “sentence cluster”**. We defined the TDF of a “word cluster” as the percentage of an amino acid cluster within the “word cluster” among its total amino acid clusters in three successive days. The mean TDF of a “word cluster” was determined by dividing the total TDF of a “word cluster” by the number of TDF. The prevalent “word clusters” were identified as clusters with a mean TDF higher than 0.2. When a prevalent “word cluster” was generated, the CF of the amino acid clusters at other “word clusters” was calculated based on the dataset. The amino acids at other “word clusters” were subsequently constrained based on the CF using the randsrc function in MATLAB R2020b.
4. **Constraint of the collocation of “sentence clusters” within each “paragraph cluster”**. The TDF of a “sentence cluster” was defined as the percentage of an amino acid cluster within a “sentence cluster” among its total amino acid clusters in three successive days. The mean TDF of a “sentence cluster” was determined by dividing the total TDF of a “sentence cluster” by the number of TDF. The prevalent “sentence cluster” was identified as the “sentence cluster” with a mean TDF higher than 0.2. When a prevalent “sentence cluster” was generated, the amino acids in other “sentence clusters” were subsequently constrained based on the CF using the randsrc function in MATLAB R2020b.

### Mutation simulation with the mutational profile

The “mutational profile”, defined as the occurrence frequency of mutations, was incorporated into the amino acid simulation model in order to simulate future mutations at the hot spots.

1. **Mutation simulation with the mutational profile**. We introduced a mutation flag to the model: the mutation occurred when the mutation flag was generated during the simulation. Thus, the mutational profile was the occurrence frequency of the mutation flag. The deletion was not considered in the mutation simulation. The more mutable hot spots were defined as those with more than one prevalent residue (mean TDF > 0.2). The mutational profile of the more mutable and other hot spots was set to a high value (0.9) and a medium value (0.5), respectively (Supplementary Fig. 28). The randsrc function in MATLAB R2020b was used to simulate the mutations at each hot spot. The input data of the randsrc function were the dimension of the output matrix (1, 1), the amino acids and mutation flag, and the occurrence frequency of the amino acids and mutation flag: randsrc(1, 1, [double(‘amino acid 1’), double(‘amino acid 2’), mutation flag; the occurrence frequency of amino acid 1, the occurrence frequency of amino acid 2, the mutational profile]). The number of simulations (num) was 10,000.
  We calculated a D-value as the difference between the last and first three days” frequency of each amino acid. The original residue at a hot spot was defined as the amino acid with a mean TDF greater than 0.02 (Supplementary Fig. 29). If more than one amino acid at a hot spot had a mean TDF greater than 0.02, the residue with a negative D-value was identified as the original residue (Supplementary Fig. 29). An available mutation was defined as a residue other than the original residue and the deletion for a site. When a mutation flag was detected, the percentage of an available mutation for a site was employed as the occurrence frequency to generate the mutation. The randsrc function in MATLAB R2020b was applied to simulate the mutations at each hot spot. The input data of the randsrc function were the dimension of the output matrix (1, 1), the available mutations, and the occurrence frequency of the available mutations: randsrc(1, 1, [double(‘available mutation 1’), double(‘available mutation 2’); the occurrence frequency of available mutation 1, the occurrence frequency of available mutation 2]). Then the amino acids were constrained based on the CF.
2. **LSTM model scoring of the mutations**. The mutations were generated at the hot spots in the mutation simulation in previous steps. Nonetheless, further evaluation was required for the combinatorial amino acids at hot spots within each “paragraph cluster” when the mutation occurred. The language model learned the occurrence frequency of a word or sentence given its sequence context of a paragraph.^21^ The Bi-directional Long Short-Term Memory (Bi-LSTM) language model could access both left and right contexts and export an emotional score of the sequences (Fig. 1b). In this study, the Bi-LSTM language models were trained based on dataset-1, dataset-2, or dataset-3 to calculate the emotional scores of the “paragraph clusters” when detecting a mutation. Following is an explanation of the modeling procedure.
  The Bi-LSTM language model architecture is shown in Fig. 1b. An input layer, two LSTM layers, and an output layer compromised the Bi-LSTM language model.^21^ The input data of the Bi-LSTM model were the amino acid clusters in each “paragraph cluster”. The input data excluded the clutters consisting of original amino acids and the clusters consisting only of deletions. The Bi-LSTM layer has two LSTM layers, accessing both left and right sequence contexts (Fig. 1b). The emotional scores for amino acid clusters with occurrence frequencies above and below 0.001 were set to 1 and 0, respectively. The output data of the Bi-LSTM model were the emotional score of each “paragraph cluster”. 5,000 epochs were trained in each Bi-LSTM language model. When detecting a mutation in the simulation process, the emotional score of the cluster was output by the trained Bi-LSTM language model. The mutations were regenerated if the “paragraph clusters” had a negative emotional score. Only “paragraph clusters” with a positive emotional score were output.
3. **Output the simulation results of the amino acids**. The amino acids at hot spots were simulated based on the “grammatical frameworks” with a mutational profile. The amino acid simulation results were output in accordance with the S1 protein site order (14^th^-685^th^).

### Bi-LSTM construction

The Bi-directional Long Short-Term Memory (Bi-LSTM) language model could access both left and right contexts and export an emotional score of the sequences. The forward and backward hidden vector sequences of the Bi-LSTM model is generated as follows:

1 $${\overrightarrow{h}}_{t}=H({w}_{x\overrightarrow{h}}{x}_{t}+{w}_{\overrightarrow{h}\overrightarrow{h}}{\overrightarrow{h}}_{t-1}+{b}_{\overrightarrow{h}})$$

2 $${\overleftarrow{h}}_{t}=H({w}_{x\overleftarrow{h}}{x}_{t}+{w}_{\overleftarrow{h}\overleftarrow{h}}{\overleftarrow{h}}_{t+1}+{b}_{\overleftarrow{h}})$$

In which ${\overrightarrow{h}}_{t}$ and $\overleftarrow{{h}_{t}}$ are the forward and backward hidden vector sequences, *H* is the activation function, *x*_*t*_ is the input layer, ${b}_{\overrightarrow{h}}$ and ${b}_{\overleftarrow{h}}$ are the forward and backward biases, ${w}_{x\overleftarrow{h}}$,${w}_{x\overrightarrow{h}}$, ${w}_{\overrightarrow{h}\overrightarrow{h}}$, and ${w}_{\overleftarrow{h}\overleftarrow{h}}$ are the weight matrices.

The final output sequence *h*_t_ can be calculated by Eq. (3):

3 $${h}_{t}=\partial {\overrightarrow{h}}_{t}+\beta {\overleftarrow{h}}_{t}$$

In which *α* and *β* are the weight matrices corresponding to the forward and backward hidden vectors.

### Spike plasmid construction and cloning

We used the full-length BA.5 sequence as our template and excluded the EcoRI (G^AATTC) and NheI (G^CTAGC) cleavages site for codon optimization. We also need to maintain the 702-704 codons to be GAGAATTCC for EcoRI cleavage. We inserted a homologous sequence on the 5’ end of the sequences, which would include an EcoRI cleavage site (GAATTC), a Kozak sequence (GCCACC) for stability, and part of the pCAGGs carrier’s sequence (CTGTCTCATCATTTTGGCAAA). Similarly, on the 3’ end, we added a NheI cleavage site (G^CTAGC) and another carrier’s sequence (AGATCTTTTTCCCTCTGCCAAAA). After the above sequences have been synthesized accordingly, via homologous recombination, we inserted this full-length BA.5 sequence into the pCAGGs carrier, a common carrier for SARS-CoV-2 variants. We obtained a new template for the later S1 protein subunit synthesis. Likewise, for each predicted S1 protein sequence, we also excluded the EcoRI (G^AATTC) and NheI (G^CTAGC) cleavages site for codon optimization. On the 5’ end, we also inserted a pCAGGs carrier’s sequence (CTGTCTCATCATTTTGGCAAA), an EcoRI cleavage site (GAATTC), and a Kozak sequence (GCCACC). Hence, we added an optimized nucleic acid sequence with 13 codons (MFVFLVLLPLVSS) for 5’ end. For the 3’ end, we kept the sequence to be the same as that of the full-length BA.5. After the sequences’ synthesis and the amplification of the S1 subunit sequence by PCR (Polymerase Chain Reaction), we used EcoRI to cleave the BA.5 plasmid and by homologous recombination we inserted the S1 subunit into the pCAGGs carrier with S2 unit on it. In such cases, we acquired plasmids with full-length spike protein that had different S1 sequences but the same S2 sequence. The advantage of this cloning protocol was that it used the autologous cleavage sequence EcoRI for cleavage and did not introduce an exogenous sequence that might influence our study result.

### Production and titration of pseudotyped variants

HEK293T cells were diluted to 2.5 × 105 cells/mL and inoculated into 6-well plates with 4 mL per well, then incubated at 37 °C with 5% CO_2_ overnight until the confluency for adherent cells reached 90%. The spike protein expression plasmids were cotransfected with the HIV-1 backbone plasmid in a ratio of 1:150 into HEK293T cells with the Turbofect transfection reagent (Thermo Scientific, Waltham, MA, USA). The supernatants containing pseudotyped viruses were collected at 24, 48, and 72 hours after transfection and centrifuged, then divided into aliquots and cryopreserved at −80 °C.

The amount of pseudotyped virus prepared was quantified using ELISA methods by the QuickTiter™ Lentivirus Titer Kit (CELL BIOLABS, VPK-107), and the pseudotyped viruses were diluted to the same concentration based on the HIV p24 level, and then were threefold serially diluted for a total of nine dilutions, each with six replicate wells. After dilution, hACE2-293T cells diluted in DMEM supplemented with 10% FBS were added at 2.5 × 104 cells/well and incubated at 37 °C and 5% CO_2_. The pseudotyped HIV-1 particles encode firefly luciferase in their lentiviral vector genome, so by measuring the fluorescence intensity, we could record the relative quantity of ACE2-bound, spike-expressed pseudovirus.^45^ After a 48 h incubation, the luciferase luminescence (RLU) was detected by a microplate luminescence detector (TECAN. SPARK 10 M) using Bright-Lite detection reagent (Vazyme, DD1204), and the 50% tissue culture infectious dose (TCID50) of the pseudotyped virus was calculated according to the Reed-Muench method.

### Pseudovirus-based neutralization assays

Pseudovirus-based neutralization assays were performed using the human immunodeficiency virus (HIV) pseudotyped virus production system. Genes encoding the full-length spike proteins of predicted variants and Omicron BA.5 variant (hCoV-19/South Africa/NICD-N35214/2022, GISAID EPI_ISL_11542270) were human codon optimized and inserted into the pCAGGS vector. HEK293T cells were inoculated into cell dishes and allowed to grow overnight at 37 °C and 5% CO_2_. The HIV-vectored pNL4-3.Luc.R-E- and spike protein-expressing plasmids were co-transfected with TurboFect transfection reagent (Thermo Scientific). Supernatants were collected 24-, 48-, and 72-hours post-transfection, filtered, aliquoted and frozen at −80 °C before use.

Neutralizing activity in each sample was measured with a serial dilution approach. Each sample was serially diluted 3-fold in duplicate from 1:30 to 1:7290 in complete DMEM before incubation with the titrated pseudovirus SARS-CoV-2 (105 RLU per well) for 1 hour prior to the addition of 2 × 104 293T-ACE2 cells. Following a 48 h incubation period at 37 °C and 5% CO_2_, luciferase activity was determined with the Brite-LiteTM Luciferase Assay System (Vazyme) using the GloMax® Navigator Microplate Luminometer (Promega). EC50 neutralization titers were calculated using the Reed-Muench method. The lower limit of detection (LLOD) was 30, and titers below the LLOD were set to 15.

### Flow cytometry

For S protein expression and conformational verification, 293 T cells were seeded into 24-well plates (Corning) and transfected with 0.5 μg of plasmids encoding full-length SARS-CoV-2 S protein or its mutants using Lipo3000 reagent (Invitrogen, L3000015). After cultivation at 37 °C for 36 h, the medium was removed, and cells were collected using PBS containing 0.02% EDTA. Cells were washed with PBS by centrifugation at 600 × g for 5 min and then incubated with 5 μg/mL fluorescein isothiocyanate (FITC) (Sigma, F4274) -labelled recombinant ACE2 protein (Sino Bioligical, 10108-H08B) at 37 °C for 1 h. Cells were washed and analyzed on a FACSCanto II flow cytometer (BD Biosciences).

### Donor vaccination and blood sampling

Fifty healthy adults were vaccinated with the aerosolized adenovirus type-5 vector-based COVID-19 vaccine (Ad5-nCoV) and were infected with SARS-CoV-2 Omicron variants around 2022/12 when the BA.5 and BQ.1 variants were dominant. Blood samples, including plasma and PBMCs, were collected at least 3 weeks after the fully recovered illustrated by the nucleic acid test (NCT). All donors provided written informed consent, and this study was approved by the Medical Ethics Committee. PBMCs were separated from blood samples using Ficoll density gradient centrifugation (Tianjinhaoyang Biological Manufacture), and the blood samples were slowly transferred above equal-volume lymphocyte separation medium. After centrifugation at 800 × g for 30 min, PBMCs were collected, washed twice with PBS, resuspended in cell freezing medium (90% FBS and 10% dimethyl sulfoxide (DMSO)), and stored at −80 °C until use.

### Phylogenetic tree construction of SARS-CoV-2 variants

The variant strains of the phylogenetic tree were selected based on their demonstrated increase in infectivity, escape ability, or both. These include eight strains with enhanced infectivity, five strains with augmented escape ability, and two strains exhibiting both attributes. The selected compared strains encompass the wild-type Wuhan-Hu-1 (WT), as well as prevalent variants including BA.1.1_UJJ91847.1, BA.2_UZT65727.1, BA.5_UXR22400.1, BF.7_UXM55527.1, BQ.1_UYI38611.1, XBB.1.5_WBA68774.1, XBB.1.9_WDB06325.1, XBB.1.16_WFD66465.1.

### Average daily prevalence of SARS-CoV-2 variants

To evaluate the prevalence of the identified pivotal mutation sites in real-world, we conducted analysis of the average daily prevalence of SARS-CoV-2 variants on a global scale. The data was sourced from the genomic reports provided by Outbreak.info (https://outbreak.info/), a platform known for its scalable and dynamic surveillance of SARS-CoV-2 variants and mutations. The prevalence of the “S:R346T, S:K444T, S:N460K” variant was calculated by dividing the number of cases of the variant by the total number of cases for each 7 days (from Jan 2020 to June 2023). The error bands show the 95% binomial proportion confidence interval calculated using Jeffrey’s interval.

### 3D structure illustration of spike protein and the structural change analysis

The protein structure information of BA.5 spike protein was retrieved from protein databank (PDB) website (https://www.rcsb.org) with PDB code 7xnq. Due to the lack of structure in furin cleavage site, the missing structure was predicted and completed by Phyre2 website (https://www.sbg.bio.ic.ac.uk/~phyre2). The completed structure was uploaded in Missense 3D (http://missense3d.bc.ic.ac.uk/) for predicting the effect of structural changes introduced by each amino acid substitution.

### Sequence alignment and Model Comparison

Sequence alignment and BLAST are based on Geneious Prime (version 2024.0.05). The EVEscape model is downloaded from the Github repository (https://github.com/OATML-Markslab/EVEscape), and the MLAEP model also from the Github repository (https://github.com/WHan-alter/MLAEP). Python 3.9 and Jupyter Notebook 9.6.4 are used to construct random generators and discover mutants from a given sequence. We choose BA.2 as the original sequence for the random generator as it is the most dominant variant during the timeframe of dataset-1, so we can compare it with sequences generated by dataset-1 by our model.

### Statistical analysis

In the present study, GraphPad Prism 8.0 was used for statistical calculations and data plotting. For infectivity experiment, TCID50 value differences between the BA.5 control and the predicted samples were evaluated by Mann‒Whitney U test. For neutralization assay, differences of EC50 were analyzed by paired-t test. All tests were two-tailed, unless otherwise indicated. We considered a threshold *p*-value < 0.05 to indicate statistical significance, and the significance values were set as *.

## Supplementary information

Supplementary figures and tables

Supplementary data 3

Supplementary data 1

Supplementary data 2

## Data Availability

The source data that support the findings of this study are upon request or available at Github repository https://github.com/athirma/SVEP.

## Materials availability

Correspondence and requests for materials should be addressed to G.C. (gongcheng@mail.tsingua.edu.cn). For unexpected missing messages, alternative contacts could be C.W. (weicongwen@aliyun.com), E.M. (meh20@mail.tsinghua.edu.cn), X.G. (15210418734@163.com), P.W. (pewang@uchc.edu).

## Code availability

The source code that support the findings of this study are available at Github repository https://github.com/athirma/SVEP.
