# Supplementary material for: A predictive language model for SARS-CoV-2 evolution
Source: Signal Transduct Target Ther. 2024 Dec 23;9:353. doi: 10.1038/s41392-024-02066-x (PMC11663983; doi:10.1038/s41392-024-02066-x)
Supplement: Supplementary file 1 — Supplementary figures and tables [file 41392_2024_2066_MOESM1_ESM.docx]

Supplementary Material for

**A Predictive Language Model For SARS-CoV-2 Evolution**

Enhao Ma ^1, #^, Xuan Guo ^1, 3, #, *^, Mingda Hu ^2^, Penghua Wang ^4^, Xin Wang ^2^, Congwen Wei ^2, *^, and Gong Cheng ^1, 3, *^

^1^ School of Basic Medical Science, Tsinghua University, 30 Shuangqing Rd., Haidian District, Beijing 100084, China

^2^ Beijing Institute of Biotechnology, 20 Dongdajie, Fengtai District, Beijing 100071, China

^3^ Institute of Infectious Diseases, Shenzhen Bay Laboratory, Guangqiao Rd., Guangming District, Shenzhen, Guangdong 518000, China

^4^ Department of Immunology, School of Medicine, University of Connecticut Health Center, Farmington, CT 06030, USA

∗ Corresponding authors.

E-mail addresses: [gongcheng@mail.tsinghua.edu.cn](mailto:gongcheng@mail.tsinghua.edu.cn). (G.C.), [weicongwen@aliyun.com](mailto:weicongwen@aliyun.com) (W. C.), [15210418734@163.com](mailto:15210418734@163.com) (X.G.)

^#^ These authors contributed equally to this work.

**This file includes:**

Supplementary Figures 1 to 29

Supplementary Tables 1 and 2

**Supplementary Fig. 1** The frequencies and counts of Omicron subvariants in dataset-1 (April 15^th^, 2022, to September 15^th^, 2022) (**a**), dataset-2 (September 16^th^, 2022, to May 10^th^, 2023) (**b**), and dataset-3 (May 15^th^, 2023, to October 31^st^, 2023) (**c**).

**Supplementary Fig. 2** Screening for the hot spots and non-hot spots based on dataset-1. **a** The dominant residue of each site of dataset-1. **b** The Three Days’ Frequency (TDF) of the dominant residue in each site of dataset-1. **c** The criterion for screening the hot spots. *y*_max_ and *y*_min_ denote the maximum and minimum TDF of the dominant amino acid of a site. *N* denotes the number of amino acids that appear in a site. **d** The hot and non-hot spots screened in dataset-1. The orange and grey solid circles denote the hot and non-hot spots, respectively. The number in each circle denotes the residue site. del denotes the amino acid deletion.

**Supplementary Fig. 3** Determination of the “word clusters” based on dataset-1. **a** Determination of the “word clusters” number by a Hierarchical cluster approach. **b** The “word clusters” determined by a K-means cluster approach. The orange solid circles denote the hot spots. The number in each circle denotes the residue site. The black dashed circles denote the “word clusters.”

**Supplementary Fig. 4** Determination of the “sentence clusters” based on dataset-1. **a** Determination of the “sentence clusters” number by a Hierarchical cluster approach. **b** The “sentence clusters” determined by a K-means cluster approach. The orange solid circles denote the hot spots. The number in each circle denotes the residue site. The black and green dashed circles denote the “word clusters” and “sentence clusters,” respectively.

**Supplementary Fig. 5** Determination of the “paragraph clusters” based on dataset-1. **a** Determination of the “paragraph clusters” number by a Hierarchical cluster approach. **b** The “paragraph clusters” determined by a K-means cluster approach. The orange solid circles denote the hot spots. The number in each circle denotes the residue site. The black, green, and blue dashed circles denote the “word clusters,” “sentence clusters,” and “paragraph clusters,” respectively.

**Supplementary Fig. 6** The “grammatical frameworks” of the sequences in dataset-1. The orange solid circles denote the hot spots. The number in each circle denotes the residue site. The black, green, and blue dashed circles denote the “word clusters,” “sentence clusters,” and “paragraph clusters,” respectively.

**Supplementary Fig. 7** The performance of model’s restoration and prediction based on dataset-1 and dataset-2. **a** The frequencies comparison of the majority of variants found in dataset-1 and restored by the simulation of the models’ “grammatical framework” constructed from dataset-1. **b** The frequencies of the majority of variants predicted by the model with a variable mutational profile based on dataset-1. **c** The frequencies comparison of the majority of variants found in dataset-2 and restored by the simulation of the models’ “grammatical framework” constructed from dataset-2.

**Supplementary Fig. 8** The Accuracy, Recall, and F1 score comparison among different screen models and Bi-LSTM (used by our model).

**Supplementary Fig. 9** The frequencies of the amino acid at some sites between May 16^th^, 2023, and July 1^st^, 2023. del denotes the amino acid deletion.

**
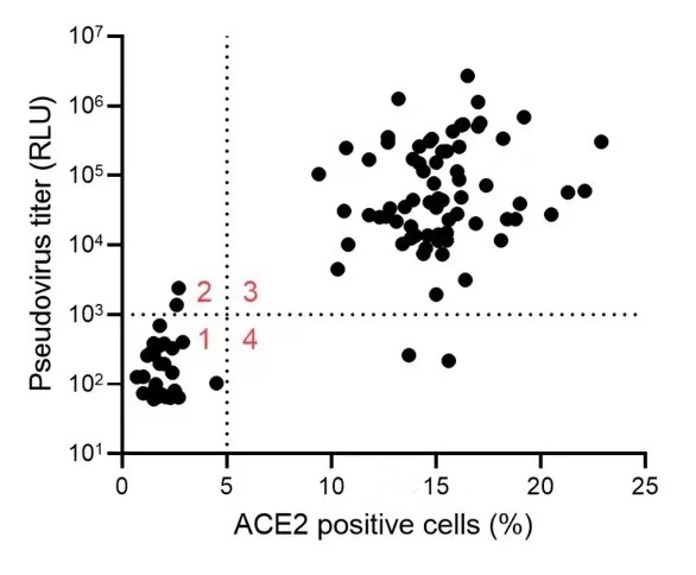
**

**Supplementary Fig. 10** The relationship between the binding affinity of predicted spike variants and hACE2 receptor proteins illustrated by the flow cytometry and the pseudovirus titer after packaging.


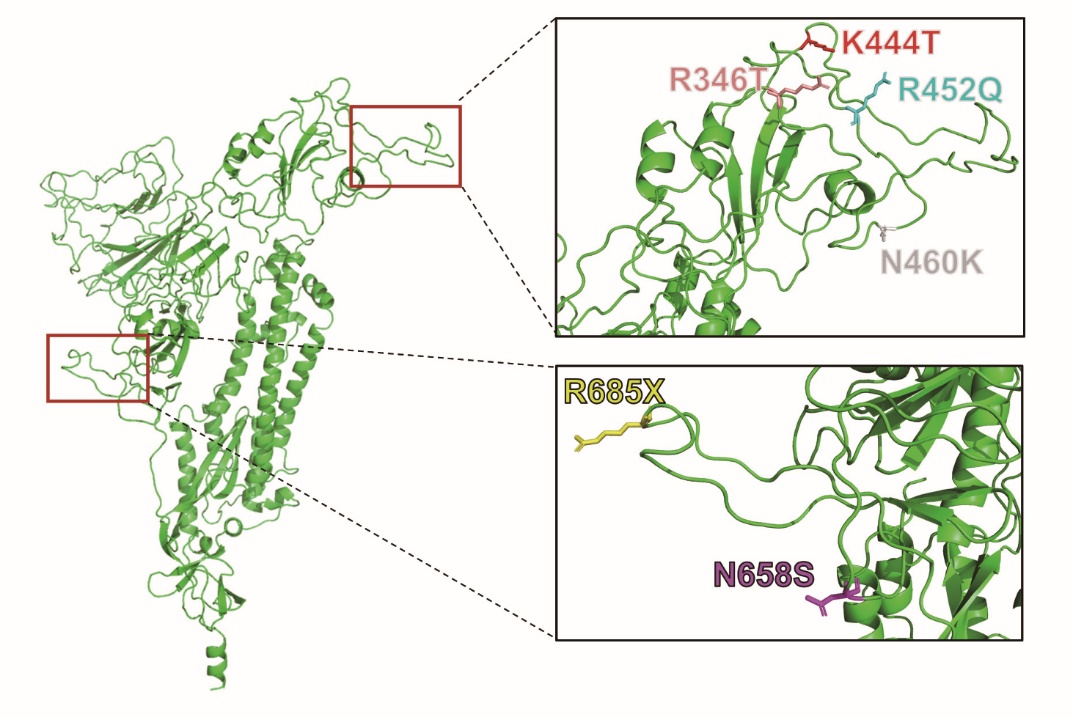


**Supplementary Fig. 11** The 3D protein structure of SARS-CoV-2 Omicron BA.5 subvariants with R346T, K444T, R452Q, N460K, R685X, and N658S mutation sites being marked.


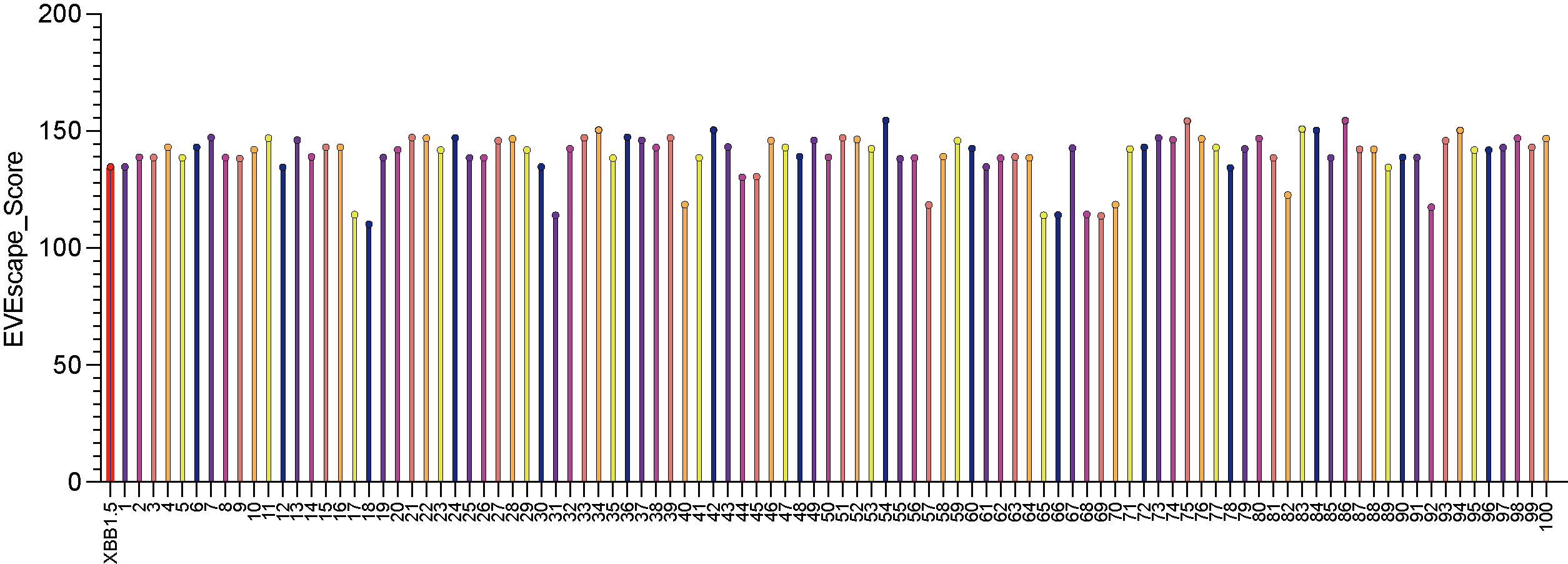


**Supplementary Fig. 12** The EVEscape scoring of the sequences predicted by our model based on dataset-2. The 100 sequences predicted by our model are based on dataset-2, and the dominant variant XBB1.5 during dataset-2 is used for comparison.


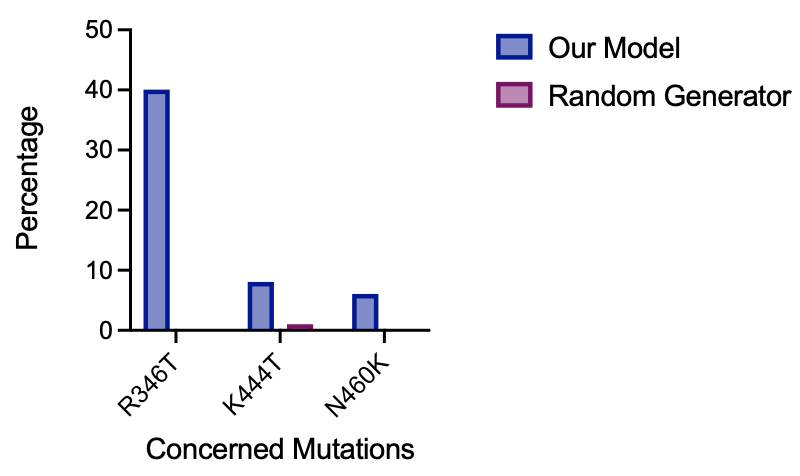


**Supplementary Fig. 13** The comparison for percentage of concerned mutation between our model and a random generator.

**Supplementary Fig. 14** Screening for the hot spots and non-hot spots based on dataset-2. **a** The dominant residue of each site of dataset-2. **b** The Three Days’ Frequency (TDF) of the dominant residue in each site of dataset-2. **c** The criterion for screening the hot spots. *y*_max_ and *y*_min_ denote the maximum and minimum TDF of the dominant residue of a site. *N* denotes the number of amino acids that appear in a site. **d** The hot and non-hot spots screened in dataset-2. The orange and grey solid circles denote the hot and non-hot spots, respectively. The number in each circle denotes the residue site. del denotes the amino acid deletion.

**Supplementary Fig. 15** Screening for the hot spots and non-hot spots based on dataset-3. **a** The dominant residue of each site of dataset-3. **b** The Three Days’ Frequency (TDF) of the dominant residue in each site of dataset-3. **c** The criterion for screening the hot spots. *y*_max_ and *y*_min_ denote the maximum and minimum TDF of the dominant residue of a site. *N* denotes the number of amino acids that appear in a site. **d** The hot and non-hot spots screened in dataset-3. The orange and grey solid circles denote the hot and non-hot spots, respectively. The number in each circle denotes the residue site. del denotes the amino acid deletion.

**Supplementary Fig. 16** Determination of the “word clusters” based on dataset-2. **a** Determination of the “word clusters” number by a Hierarchical cluster approach. **b** The “word clusters” determined by a K-means cluster approach. The orange solid circles denote the hot spots. The number in each circle denotes the residue site. The black dashed circles denote the “word clusters.”

**Supplementary Fig. 17** Determination of the “word clusters” based on dataset-3. **a** Determination of the “word clusters” number by a Hierarchical cluster approach. **b** The “word clusters” determined by a K-means cluster approach. The orange solid circles denote the hot spots. The number in each circle denotes the residue site. The black dashed circles denote the “word clusters.”

**Supplementary Fig. 18** Determination of the “sentence clusters” based on dataset-2. **a** Determination of the “sentence clusters” number by a Hierarchical cluster approach. **b** The “sentence clusters” determined by a K-means cluster approach. The orange solid circles denote the hot spots. The number in each circle denotes the residue site. The black and green dashed circles denote the “word clusters” and “sentence clusters,” respectively.

**Supplementary Fig. 19** Determination of the “sentence clusters” based on dataset-3. **a** Determination of the “sentence clusters” number by a Hierarchical cluster approach. **b** The “sentence clusters” determined by a K-means cluster approach. The orange solid circles denote the hot spots. The number in each circle denotes the residue site. The black and green dashed circles denote the “word clusters” and “sentence clusters,” respectively.

**Supplementary Fig. 20** Determination of the “paragraph clusters” based on dataset-2. **a** Determination of the “paragraph clusters” number by a Hierarchical cluster approach. **b** The “paragraph clusters” determined by a K-means cluster approach. The orange solid circles denote the hot spots. The number in each circle denotes the residue site. The black, green, and blue dashed circles denote the “word clusters,” “sentence clusters,” and “paragraph clusters,” respectively.

**Supplementary Fig. 21** Determination of the “paragraph clusters” based on dataset-3. **a** Determination of the “paragraph clusters” number by a Hierarchical cluster approach. **b** The “paragraph clusters” determined by a K-means cluster approach. The orange solid circles denote the hot spots. The number in each circle denotes the residue site. The black, green, and blue dashed circles denote the “word clusters,” “sentence clusters,” and “paragraph clusters,” respectively.

**Supplementary Fig. 22** The “grammatical frameworks” of the sequences in dataset-2. The orange solid circles denote the hot spots. The number in each circle denotes the residue site. The black, green, and blue dashed circles denote the “word clusters,” “sentence clusters,” and “paragraph clusters,” respectively.

**Supplementary Fig. 23** The “grammatical frameworks” of the sequences in dataset-3. The orange solid circles denote the hot spots. The number in each circle denotes the residue site. The black, green, and blue dashed circles denote the “word clusters,” “sentence clusters,” and “paragraph clusters,” respectively.

**Supplementary Fig. 24** The flow chart for the Monte Carlo simulation based on the “grammatical frameworks”.

**Supplementary Fig. 25** The mean Three Days’ Frequency (TDF) of the dominant residues at each hot spot of dataset-1 (**a**), dataset-2 (**b**), and dataset-3 (**c**).

**Supplementary Fig. 26** The prevalent residue at each hot spot of dataset-1 (**a**), dataset-2 (**b**), and dataset-3 (**c**). del denotes the amino acid deletion.

**Supplementary Fig. 27** The schematic diagram for constraining the collocations of amino acids within each cluster. del denotes the amino acid deletion.

**Supplementary Fig. 28** The flow chart for modeling the future sequences by introducing a variable mutational profile.

**Supplementary Fig. 29** The original residue at each hot spot within each “paragraph cluster” of dataset-1 (**a**), dataset-2 (**b**), and dataset-3 (**c**). del denotes the amino acid deletion.

**Supplementary Table 2** The wet-lab experiment performed by all models

**Supplementary Table 1** The requirement and features comparison of other models and our model SVEP.

**Supplementary Data 1** BA.5 neutralization assay raw data

See separate file “BA.5.xlsx”

**Supplementary Data 2** Comparison with Brian's LSTM model

See separate file “Brians LSTM.xlsx”

**Supplementary Data 3** Comparison with EVEscape model

See separate file “all_seqs_EVEscape_scores-10000.csv”
